# Association of follicular helper T and follicular regulatory T cells with severity and hyperglycemia in hospitalized COVID-19 patients

**DOI:** 10.1080/21505594.2022.2047506

**Published:** 2022-03-14

**Authors:** Asmaa M. Zahran, Mona H. Abdel-Rahim, Khalid A. Nasif, Safinaz Hussein, Rania Hafez, Ahmad Bahieldeen Ahmad, Khaled Saad, Amira Elhoufey, Hosni A. M. Hussein, Ali A. Thabet, Omnia El-Badawy

**Affiliations:** a Department of Clinical Pathology, South Egypt Cancer Institute, Assiut University, Assiut, Egypt; b Department of Medical Microbiology and Immunology, Faculty of Medicine, Assiut University, Assiut, Egypt; c Department of Medical Biochemistry, Faculty of Medicine, Minia University, Minia, Egypt; d Department Clinical Biochemistry, College of Medicine, King Khalid University,Abha, Saudi Arabia; e Department of Internal Medicine, Clinical Hematology Unit, Faculty of Medicine, Assiut University, Assiut, Egypt; f Department of Internal Medicine, Critical Care Unit, Faculty of Medicine, Assiut University, Assiut, Egypt; g Department of Pediatrics, Faculty of Medicine, Assiut University, Assiut, Egypt; h Department of Community Health Nursing, Faculty of Nursing, Assiut University, Assiut, Egypt; I Department of Community Health Nursing, Alddrab University College, Jazan University, Jazan, Saudi Arabia; j Department of Microbiology, Faculty of Science, Al Azhar University, Assiut 71524, Egypt; k Department of Zoology, Faculty of Science, Al Azhar University, Assiut 71524, Egypt

**Keywords:** COVID-19, follicular regulatory T, follicular helper T, hyperglycemia

## Abstract

We aimed to determine the levels of follicular helper T (Tfh) and follicular regulatory T (Tfr) cells in COVID-19 patients and determine whether their levels correlated with disease severity and presence of hyperglycemia. This study was carried out in 34 hospitalized COVID-19 patients and 20 healthy controls. Levels of total circulating Tfh, inducible T-cell costimulator (ICOS)+ activated Tfh, and Tfr cells were assessed in all participants by flow cytometry. Total CD4+CXCR5+ Tfh cells and ICOS+Foxp3-activated Tfh cells increased and ICOS+Foxp3+ Tfr cells decreased in COVID-19 patients, especially in diabetic patients and those with severe disease. Activated ICOS+ Tfh cells were directly correlated with lactate dehydrogenase, D-dimer, ferritin, and respiratory rate and inversely correlated with the partial pressure of carbon dioxide. COVID-19 is associated with marked activation of Tfh cells and a profound drop in Tfr cells, especially in severe and diabetic patients. Future studies on expanded cohorts of patients are needed to clarify the relationship between SARS-CoV-2 and acute-onset diabetes.

## Introduction

A new coronavirus strain (SARS-CoV-2) caused an outbreak of severe pneumonia in China, in 2019. In February 2020, WHO called the disease caused by this new coronavirus COVID-19 and later announced that COVID-19 outbreak was classified as pandemic [1, [2]].

The pathophysiology of the remarkably contagious disease COVID-19 remains obscure [3]. Several immune mechanisms, in coordination with defense mechanisms, are essential for eliminating viral infections. Immune responses mediated by T cells are critical in controlling viral infections. Upon viral infection, strong type I inflammation promotes the virus-specific CD4+ T cell differentiation into primarily T helper 1 (Th1) and follicular helper T cells (Tfh) [4].

Tfh cells are featured by the high expression of the chemokine receptor C-X-C motif chemokine receptor 5 (CXCR5) [5]. Likewise, an inducible T cell costimulator (ICOS) is essential for production and maintenance of Tfh cells [6]. The production of neutralizing antibodies (nAbs) in the germinal center (GC) requires Tfh cell help; therefore, these cells represent a major line of defense to pathogens [7]. They are specialized in supporting B cell survival, differentiation, and proliferation within the GCs in the follicles of secondary lymphoid organs via direct contact and secretion of soluble signals [8].

Follicular regulatory T cells (Tfr) are a subgroup of regulatory T cells (Tregs) derived mainly from thymic Tregs [9]. They possess features of traditional Tregs and Tfh cells, as they concurrently express markers of Tregs including cytotoxic T lymphocyte antigen-4 (CTLA-4), forkhead box protein 3 (Foxp3) and, interleukin-10 (IL-10), and markers of Tfh including ICOS, CXCR5 and programmed cell death protein 1 (PD-1) [10]. They play an essential regulatory role in GC. Tfr cells inhibit Tfh and B lymphocytes, thus suppressing humoral immunity [9]. Moreover, Tfr cells secrete granzyme B, which mediates B cell and Tfh cell death [11].

Tfh and Tfr exert opposing regulatory actions in GC responses, and an equilibrium of their functions is essential for maintaining immune homeostasis [12]. Moreover, animal experiments have revealed that neutralizing antibodies (nAbs) are critical components of protective immune responses in infection and following vaccination [13]. Therefore, a better understanding of the role of Tfh and Tfr in the formation of nAbs is fundamental for the design of future vaccines.

The development of severe COVID-19 has been associated with several comorbidities. Therefore, studying the relationship between immune cells and different severity markers could be of utmost importance in the early and prompt identification and control of severe cases [12]. Diabetes is the most recorded comorbidity in patients with COVID-19. SARS-CoV-2 might increase the risk of hyperglycemia in patients with and without a prior history of diabetes. However, it is not clear whether the virus can induce type-1 or type-2 diabetes or alternatively causes a new atypical form of diabetes. It’s also unknown if recovering COVID-19 patients experience a higher risk of developing new-onset diabetes or its complications going forward [14].

Data about the alterations of Tfh and Tfr in SARS-CoV-2 infection, especially in diabetic patients are so far limited. Thus, we aimed to determine the levels of follicular helper T (Tfh) and follicular regulatory T (Tfr) cells in COVID-19 patients and determine whether their levels correlated with disease severity and presence of hyperglycemia.

## Materials and methods

Our study was approved by ethical committee of Assiut University (IRB no. 17300436). All participants provided informed written consent.

**Figure 1.**
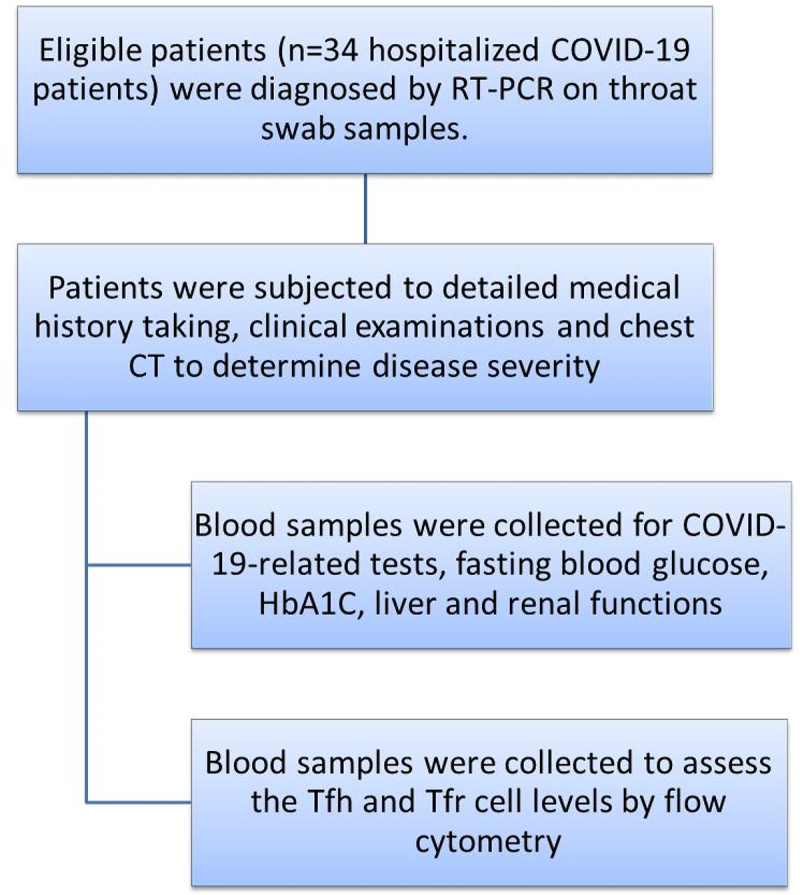
A schematic flow chart of the study protocol.

This case-control study was conducted in a sample of 34 hospitalized COVID-19 patients and 20 age- and sex-matched healthy controls. The diagnosis of COVID-19 was based primarily on reverse-transcription polymerase chain reaction (RT-PCR) on throat swab samples. All healthy controls were clinically free of disease and showed no evidence of infection by history, clinical examination, and complete blood count (CBC). In addition, none of the controls had a history of close contact with a COVID-19–positive patient in the two weeks preceding the study. A schematic flow chart of the study protocol is presented in Figure 1.

Patients were subjected to a collection of a detailed medical history and clinical examinations. Laboratory tests, including CBC and COVID-19-related tests such as D-dimer, ferritin, CRP, and LDH, were performed for all participants. Oxygen saturation (SpO_2_) and partial pressure of carbon dioxide (PaCO_2_) were measured. Fasting blood glucose (FBG), hemoglobin A1C (HbA1C), renal function (serum urea and creatinine), and liver function (albumin, total protein, direct and total bilirubin, prothrombin time, and concentration) were also measured. Diagnosis of pneumonia was based on respiratory and systemic symptoms and clinical signs (fever, cough, dyspnea, rapid shallow breathing), as well as radiography. Severe cases were characterized as those who had clinical signs of pneumonia and met at least one of the following criteria: severe respiratory distress, tachypnea >30 breaths/min, SpO2 < 90%, or greater than 50% lung infiltrates (as evident from CT) [15–16]. For all participants, blood samples were collected into EDTA-containing vacutainer tubes nearly 10–15 days from diagnosis to assess the Tfh and Tfr cells.

**Figure 2.**
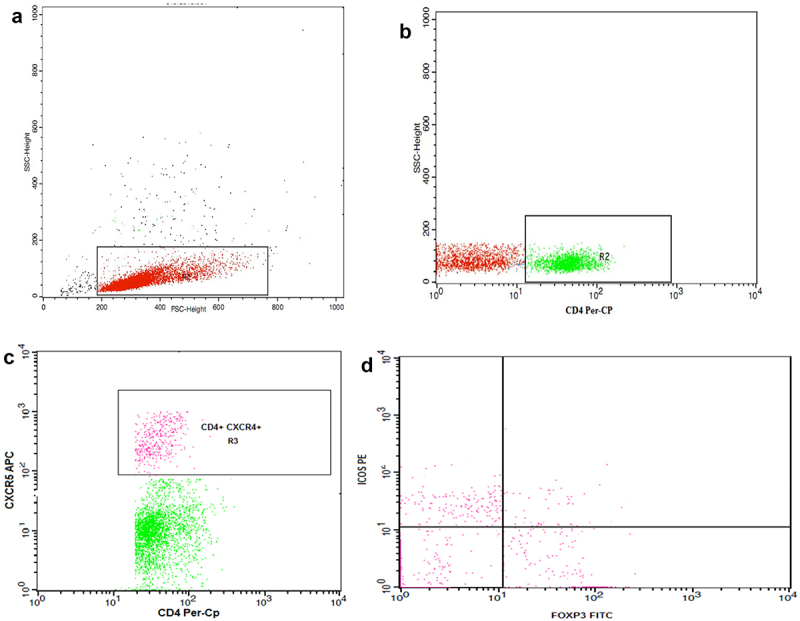
Flow cytometry gating strategy of follicular helper T cells (Tfh) and follicular regulatory T cells (Tfr) using FACSDiva software (Becton Dickinson Biosciences, USA).

Approximately 1 × 10^6^ cells in 100 µl were stained with 10 µl of phycoerythrin (PE)-conjugated anti-ICOS (R&D Systems, USA), peridinium-chlorophyll-protein (Per-CP)-conjugated anti-CD4 (R&D Systems, USA), and allophycocyanin (APC)-conjugated anti-CXCR5 (R&D Systems, USA) monoclonal antibodies for 20 min. Next, RBC lysis, wash with phosphate-buffered saline (PBS), adding of fixation solution, and incubation for 10 min were performed. After the cells were again washed with PBS, and the permeabilizing solution and 10 µl of fluoroisothiocyanate (FITC)-conjugated anti-Foxp3 (eBioscience, USA) were added and incubated for 20 min. The cells were resuspended in PBS following washing. Anti-human IgG was used as isotype-matched negative control for every sample. A total of 50,000 events were acquired and analyzed, as shown in Figure 2, using FACSCanto and FACSCalibur flow cytometers (Becton Dickinson Biosciences, USA). Tfh cells were identified as CD4+CXCR5+ T lymphocytes, activated Tfh cells as CD4+CXCR5+ICOS+Foxp3-, resting Tfh cells as CD4+CXCR5+ICOS-Foxp3-, and Tfr cells as CD4+CXCR5+ICOS+Foxp3+.

(a) Lymphocytes were gated (R1) according to their forward and side scatter properties. (b, c) CD4+ T cells were then selected by drawing (R2), then (R3) was set to identify the CD4+ T cells expressing CXCR5 (Tfh). (d) A dot plot showing the activated Tfh cells (CD4+CXCR5+ICOS+Foxp3-), resting Tfh (CD4+CXCR5+ICOS-Foxp3-), and Tfr cells (CD4+CXCR5+ICOS+Foxp3+).

## Statistical analysis

Data are expressed as mean and standard error. The Mann–Whitney U test was used to compare the non-parametric groups, and the Spearman rank-order correlation was employed to evaluate the association between different variables. *p*-value was considered significant if <.05.

## Results

The main features of COVID-19 patients are displayed in Table 1. The patients’ mean age was 61.7 ± 2 years, and 21 patients (61.8%) were female. Most of the patients had dyspnea (26/34, 76.5% of the patients), fever (25/34, 73.5%), cough (26/34, 76.5%), and diarrhea (23/34, 67.6%). Sore throat, myalgia, and fatigue were observed in 11 (32.4%), 13 (38.2%), and 18 (52.9%) of the patients, respectively.

**Table 1.** Demographic, clinical and laboratory features of the COVID-19 patients

Parameter	Patients (*n* = 34)
Demographic data	
Age*	61.7 ± 2
Sex Male	13 (38.2%)
Female	21 (61.8%)
Clinical findings	
Dyspnea	26 (76.5%)
Fever	25 (73.5%)
Cough	26 (76.5%)
Diarrhea	23 (67.6%)
Headache	7 (20.6%)
Myalgia	13 (38.2%)
Fatigue	18 (52.9%)
Anorexia	8 (23.5%)
Sore throat	11 (32.4%)
Severity	
Non-severe	20 (59%)
Severe	14 (41%)
Diabetic patients	23 (67.6%)
FBG (70–99 mg/dl)	222.8 ± 17
−diabetic patients	273 ± 20
-non-diabetic patients	129.5 ± 8
HA1c (4–5.6%)	6.7 ± .2
CBC findings*	
TLC (4-11X10^9^/L)	11.6 ± 1
Neutrophil count (2–7 ×10^9^/L)	10.6 ± 1
Lymphocyte count (1–4.8 ×10^9^/L)	2 ± .4
COVID-19 related tests*	
CRP (up to 1 mg/dl)	63.5 ± 9
Ferritin (22–322 ng/ml)	843.4 ± 156
D dimer (up to .55 mg/L)	2.8 ± .5
LDH (100–190 U/L)	427.2 ± 34
Renal functions*	
Urea (2.5–7.1 µmol/L)	12.2 ± 2
Creatinine (44.2–97 µmol/L)	138.6 ± 25
Liver functions*	
ALT (0–45 U/L)	33.1 ± 3.4
AST (0–34 U/L)	38.5 ± 3
Total protein (64–83 g/L)	57 ± 2
Albumin (34–50 g/L)	29.5 ± 1
Total bilirubin (0–34 µmol/L)	19.9 ± 4
Direct bilirubin (0–3.4 µmol/L)	8.2 ± 2
Prothrombin time (11–14 sec)	13.4 ± .4
Prothrombin concentration (70–130%)	84.6 ± 3
Blood gases*	
SpO_2_ (>95%)	84.5 ± 2
PaCO_2_ (35–45 mmHg)	33.2 ± 2
Respiratory rate (13–28/min) *	32.4 ± 2

Data were presented as number (percentage) or *mean ± SE (normal reference ranges.

FBG fasting blood glucose, CBC complete blood count, TLC total leukocyte count, CRP C-reactive protein, LDH lactate dehydrogenase, ALT alanine aminotransferase, AST aspartate aminotransferase, SpO_2_ Oxygen saturation, PaCO_2_ partial pressure of carbon dioxide.

Approximately 59% of the participating patients had a non-severe COVID, whereas 41% had severe cases. Overall, the group of COVID patients showed a very high mean FBG (222.8 mg/dl), and 23 (67.6%) of the patients were diabetic. All but one non-diabetic patient had a higher FBG than average, with a mean of 129.5 ± 8 mg/dl. The average HbA1c level was 6.7% ± .2%, and 36% of the severe cases had uncontrolled diabetes, with HbA1c > 7%. The mean TLC was 11.6 ± 1 × 10^9^/L – levels above 10.6 x 10^9^/L indicate neutrophilia – and the mean lymphocyte count was 2 ± .4 × 10^9^/L. In severe cases, the TLC reached 12.7 ± 4 × 10^9^/L (notably, numbers below .7 × 10^9^/L indicate lymphopenia). CRP, ferritin, D dimer, and LDH were markedly elevated. Renal functions were noticeably impaired, and the liver function tests showed hypoalbuminemia and elevated direct bilirubin level (8.2 ± 2 µmol/L). The mean prothrombin time and concentration were within the normal ranges; however, in severe cases, prothrombin time reached 15 ± .9 sec. The respiratory rate was increased, reaching 40.4 ± 4 in severe cases; in addition, decreased oxygen saturation (84.5% ± 2%) and PaCO_2_ (27.4 ± 1 mmHg) were observed.

**Figure 3.**
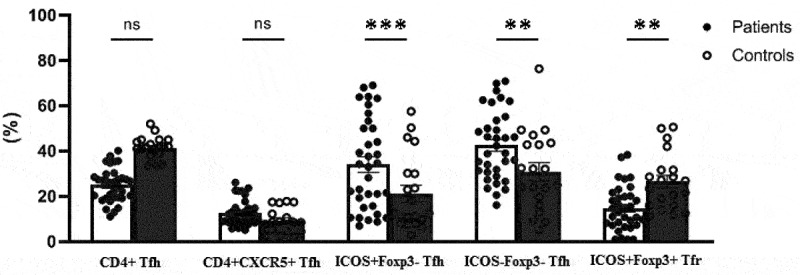
Comparison of the frequencies of follicular helper (Tfh) and follicular regulatory T (Tfr) cells between COVID-19 patients and controls (*p-*value*** <.0001, ** <.01, ns=not significant). Percentages of ICOS+ and ICOS- cells were calculated from CD4+CXCR5+ Tfh cells.

The CD4+ cells were significantly lower, while the total number of CD4+CXCR5+ Tfh cells was significantly higher in COVID-19 patients compared to controls (*p* < .0001 and *p* = .03, respectively). Levels of CD4+CXCR5+ICOS+Foxp3-activated Tfh and CD4+CXCR5+ICOS-Foxp3-resting Tfh were significantly increased in patient group (*p* = .02 and *p* = .02, respectively). In contrast, CD4+CXCR5+ICOS+Foxp3+ Tfr cells were reduced in patients compared to the controls (*p* = .04). These results are shown in Figure 3.

**Figure 4.**
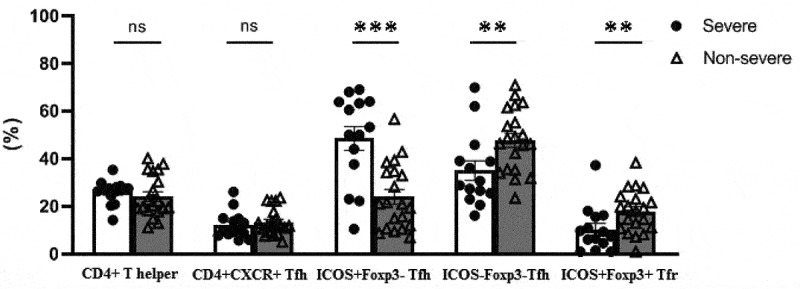
Comparison of the percentages of follicular helper (Tfh) and follicular regulatory T (Tfr) cells between severe and non-severe cases of COVID-19 (*p-*value: *** <.0001, ** <.01, ns=not significant). Percentages of ICOS+ and ICOS- cells were calculated from CD4+CXCR5+ Tfh cells.

The levels of CD4+ T helper and Tfh cells were similar in the severe and non-severe COVID-19 patients, as shown in Figure 4 The activated CD4+CXCR5+ICOS+Foxp3-Tfh cells were significantly elevated in severe COVID-19 patients compared to patients with non-severe disease (*p* < .0001). In contrast, the levels of resting CD4+CXCR5+ICOS-Foxp3- Tfh cells and CD4+CXCR5+ICOS+Foxp3+ Tfr were decreased in severe patients compared to non-severe patients (*p* = .009 and *p* = .009, respectively).

**Figure 5.**
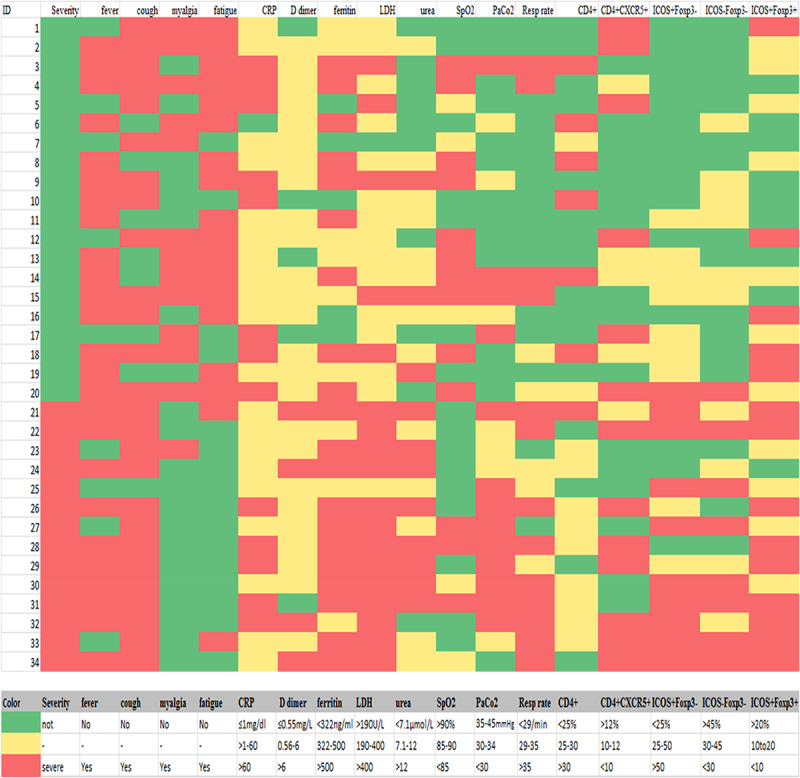
A heat map showing the levels of activated Tfh cells (Cd4+cxcr5+icos+foxp3-), resting Tfh (Cd4+cxcr5+icos-Foxp3-), and Tfr cells (Cd4+cxcr5+icos+foxp3+) in relation with clinical and laboratory findings in COVID-19 patients. as shown, most of the CD4+CXCR5+ Tfh cells were activated (Icos+) in severe cases and the increase in its level was associated with higher LDH, D-dimer, and ferritin, as well as respiratory rate, and lower PaCo_2_. Resting Tfh and Tfr cells showed opposite relations with the laboratory indices of severity.

Moreover, an inverse relationship was observed between the CD4+CXCR5+ICOS+Foxp3- activated Tfh and CD4+CXCR5+ICOS+Foxp3+ Tfr cells (*r* = > −.6, *p <* .0001). The percentage of activated CD4+CXCR5+ICOS+Foxp3-Tfh cells was directly correlated with LDH (*r* = .4, *p=*.009), D-dimer (r = .3, *p=*.04), ferritin (*r* = .3, *p=*.04), urea (*r* = .3, *p=*.02), and the respiratory rate (*r* = .7, *p* < .0001) and inversely correlated with the PaCO_2_ (*r *= > −0.5, *p* = .002). The relationships between resting Tfh and Tfr subsets, as well as clinical and laboratory data, were the inverse of those observed with activated Tfh. CD4+CXCR5+ICOS-Foxp3- Tfh and CD4+CXCR5+ICOS+Foxp3+ Tfr cells were inversely related to the LDH level (*r* = > −0.3, *p* = .02 and *r* = −.4, *p* = .01, respectively). The CD4+CXCR5+ICOS-Foxp3-Tfh cells were also negatively correlated with the serum levels of D-dimer (*r* = > −0.3, *p* = .045) and ferritin (*r* = > −0.3, *p* = .03). Additionally, the CD4+CXCR5+ICOS-Foxp3- Tfh cells were positively related to the PaCO_2_ (*r *= .4, *p=*.006) and inversely related to the respiratory rate (*r *= > −0.6, *p=*.001), Figure 5.

**Figure 6.**
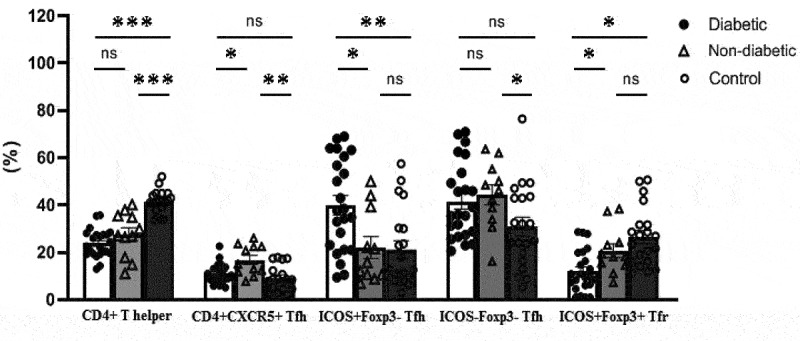
Comparison of the percentages of follicular helper (Tfh) and follicular regulatory T (Tfr) cells between diabetic and non-diabetic cases of COVID-19 and healthy controls (*p-*value: *** <.0001, ** <.01, ns=not significant). Percentages of ICOS+ and ICOS- cells were calculated from CD4+CXCR5+ Tfh cells.

As shown in Figure 6, levels of total CD4+CXCR5+ Tfh cells were significantly increased in the non-diabetic COVID-19 patients compared with healthy volunteers (*p* = .003) and diabetic COVID-19 patients (*p* = .01). In contrast, the frequency of CD4+CXCR5+ICOS+Foxp3-activated Tfh cells was remarkably increased in diabetic patients than control group and non-diabetic COVID patients. A lower frequency of CD4+CXCR5+ICOS+ Foxp3+ Tfr cells was found in the diabetic patients compared to controls (*p* = .01) and non-diabetic COVID-19 patients (*p* = .02).

## Discussion

In the mouse models infected with SARS-CoV-2, CD4+ T-cell responses showed a critical role in limiting infection [17]. Following viral infection, Tfh cells promote the production of specific nAbs by forming long-lasting memory B lymphocytes and plasma cells [18]. Circulating Tfh (cTfh) cells were found to have a similar phenotype and function to the bona fide counterparts in GCs in secondary lymphoid tissues [19].

We assessed the levels of Tfh and Tfr cells in hospitalized COVID-19 patients and healthy controls. Despite the profound reduction observed in CD4+ percentage, substantial rises were detected in the frequencies of both activated (ICOS+) and resting (ICOS-) CD4+CXCR5+ Tfh.

Consistent with published reports in acute infections, higher levels of cTfh were recently found in patients with COVID-19, and these levels rose throughout the disease [20–22]. Thevarajan et al. reported an increased frequency of Tfh in acute SARS-CoV-2 infection [21]. Others discovered that nAbs specific for SARS-CoV-2 were detectable approximately 10–15 days after COVID-19 onset [23–26]. Additionally, previous studies have demonstrated a correlation between Tfh cell frequency and antibody levels in recovering COVID-19 patients [27–29]. Juno and colleagues recently demonstrated that viral spike glycoprotein (S)–specific memory B-cells and circulating Tfh were constantly produced SARS-CoV-2 patients and were related to nAb production, indicating strong humoral immune responses [23].

Levels of CD4+ T helper and total Tfh cells in our study were almost the same in both the severe and non-severe cases. While most of the CD4+CXCR5+ Tfh cells were activated (ICOS+) in severe cases, a higher proportion of Tfh cells were resting (ICOS-) in non-severe cases. Oja and colleagues stated that the highest antibody titers and elevated frequencies of PD-1+CXCR5+ cTfh were detected in critical COVID 19 patients [30]. Additionally, Gong *et al*. found that patients with severe disease had a higher frequency of effector-memory cTfh cells and a lower frequency of central-memory cTfh cells than controls and non-severe disease patients [29].

On the other hand, Zhang and colleagues detected no difference in the frequency of total circulating Tfh cells between COVID-19-convalescent individuals and healthy controls. Meanwhile, they reported lower expression of CXCR3+ in total Tfh cells of both severe and non-severe COVID-19-convalescent individuals compared with the healthy controls. Additionally, the severe group showed a higher percentage of CXCR3+ Tfh cells than the non-severe group. CXCR3+ Tfh cells also positively correlated with neutralizing antibody titers proposing a propable key role of CXCR3+ Tfh cells in the early production of neutralizing antibody titer in patients recovering from COVID-19 [31].

Also, defective generation of Bcl-6+ Tfh cell and dysregulated humoral immune induction early in COVID-19 disease was reported in another study, providing a mechanistic explanation for the limited durability of antibody responses in coronavirus infections, and suggest that achieving herd immunity through natural infection may be difficult [32]. Further studies on Tfh subsets and Bcl-6 expression shall provide insight into their role in control of SARS-CoV2 infection.

In contrast, significantly lower percentage of Tfr cells was detected in our COVID-19 patients compared with healthy volunteers, especially in those with severe cases. Tfr cells can inhibit humoral immunity mediated by Tfh cells, constraining the GC response needed for high-affinity antibody production [9]. Along this line, our results showed an inverse relationship between the CD4+CXCR5+ICOS+Foxp3-activated Tfh and CD4+CXCR5+ICOS+Foxp3+ Tfr cells.

Data on the levels of circulating Tfr in patients with COVID-19 is scarce. Gong et al. [29] reported a reduced frequency of circulating Tfr cells (cTfr) in COVID-19 convalescent patients, with a similar decline observed in those recovering from non-severe and severe disease. They also found inverse relationships between cTfr cells and COVID-19 IgM and IgG titers.

In our patients, activated ICOS+ Tfh cells were directly correlated with LDH, D-dimer, and ferritin, as well as respiratory rate, and an inverse correlation with the PaCO_2_. Relationships between the resting Tfh and the Tfr subsets and the severity, clinical, and laboratory data were opposite to those observed for activated Tfh cells. This shows that these factors may have an impact on circulating activated Tfh and Tfr homeostasis, which may affect the subsequent antibody production.

Whereas we did not find an association between any of these cells and SpO_2_ [29], observed that the frequency of central memory cTfh cells was positively related to PaO_2_/FiO_2_ and that effector memory cTfh cells were negatively associated with PaO_2_/FiO_2_, with no strong association observed between PaO_2_/FiO_2_ and cTfr cells.

Our COVID patients had a remarkably high mean FBG, and 23 of them had diabetes. Furthermore, all but one non-diabetic patient had a higher FBG than average. Infection tends to increase stress-induced insulin resistance, leading to elevated blood glucose levels even in non-diabetic patients [33]. Numerous studies have described COVID-19 patients are at increased danger of developing glucose metabolic disorders. COVID-19 patients with hyperglycemia may be at a higher risk of more severe outcomes and death from COVID-19 even if they do not have preexisting diabetes. Serious metabolic complications of diabetes have been reported in COVID-19 patients, as diabetic ketoacidosis and probable new-onset diabetes [34,35].

SARS-CoV-2, like other coronaviruses, binds to angiotensin-converting enzyme 2 (ACE2) receptors, which are expressed in a variety of tissues, including pancreatic cells [36]. Fasting hyperglycemia and acute-onset diabetes were found to be more prevalent in patients with SARS-CoV-1 pneumonia than in those with non-SARS pneumonia. These findings suggest that COVID-19 may have a diabetogenic effect independent of the stress response observed during severe illness. However, it is unknown whether these diabetogenic effects will subside or persist following infection recovery [37].

While the available data have not established an association between SARS-CoV-2 exposure and new-onset diabetes [38], hypothesized that SARS-CoV-2 exposure accelerates the onset of type 1 diabetes. They noticed a rise in new cases of type 1 diabetes in children through the pandemic’s peak years, and some of these children had active COVID or had a history of prior exposure to the virus [38].

Knowledge of the opposite roles of Tfh and Tfr cells in regulating GC responses, maintaining immune homeostasis requires a balance of their actions [12]. Additionally, uncontrolled Tfr or Tfh activity might lead to the production of autoreactive B cells and auto-antibodies [39]. Interestingly, our results showed that diabetic COVID-19 patients demonstrated a notable rise in the frequency of activated Tfh and a profound drop in Tfr percentage compared with both the controls and non-diabetic patients. We postulate that the Tfh/Tfr imbalance observed in COVID-19 may be implicated in the complex pathogenesis of COVID-related diabetes. However, our study has some limitations. Specifically, the age and the use of some immune-modulating agents for COVID-19, such as steroids, tocilizumab (an IL-6 receptor antagonist), and COVID-19 convalescent plasma, may have confounded the findings. Also, assessment of Tfh subsets, Bcl-6 expressing Tfh and levels of neutralizing antibodies might give a clearer insight on their role in COVID-19 pathogenesis and their relation with recovery and hyperglycemia development. It would have added a lot if Tfh and Tfr levels were also assessed in a group of diabetic non-COVID patients.

## Conclusion

Our results suggest that COVID-19 is associated with marked activation of Tfh cells and a profound drop in Tfr cells, especially in diabetic patients and patients with severe disease. Future studies on expanded cohorts of patients are needed to clarify the relationship between SARS-CoV-2 and acute-onset diabetes.

## Supplementary Material

Supplemental Material

## Data Availability

The datasets generated during and/or analyzed during the current study are available from the corresponding author on reasonable request after taking a permission from our ethical committee.
