# Supplementary material for: Association of follicular helper T and follicular regulatory T cells with severity and hyperglycemia in hospitalized COVID-19 patients
Source: Virulence. 2022 Mar 14;13(1):569–77. doi: 10.1080/21505594.2022.2047506 (PMC8928811; doi:10.1080/21505594.2022.2047506)
Supplement: Supplemental Material [file KVIR_A_2047506_SM6815.docx]

**Data Availability Statements**

The datasets generated during and/or analyzed during the current study are available from the corresponding author on reasonable request after taking a permission from our ethical committee
